# CT-guided percutaneous drilling is a safe and reliable method of treating osteoid osteomas

**DOI:** 10.1186/2193-1801-2-34

**Published:** 2013-01-31

**Authors:** Edgard Eduard Engel, Nelson Fabrício Gava, Marcello Henrique Nogueira-Barbosa, Filipe Almeida Botter

**Affiliations:** 1 Department of Orthopedics and Traumatology, School of Medicine of Ribeirão Preto, University of São Paulo, Hospital das Clínicas 11° andar, Av. Bandeirantes, 3900, Monte Alegre, Ribeirão Preto, SP 14049-900 Brazil; 2 Department of Radiology, School of Medicine of Ribeirão Preto, University of São Paulo, Hospital das Clínicas 11° andar, Av. Bandeirantes, 3900, Monte Alegre, Ribeirão Preto, SP 14049-900 Brazil

**Keywords:** Osteoid osteoma, Interventional radiography, Bone remodeling, Treatment outcome, Bone neoplasms

## Abstract

Computed tomography (CT)-guided percutaneous drilling is an alternative for osteoid osteoma treatment. This study aims to evaluate the remodeling of the drill orifice. The success rate and complications were also recorded and compared with other treatment methods.

Fifteen patients with an average age of fourteen years (ranging from 4 to 25) submitted to CT-guided percutaneous drilling between 2003 and 2009 were retrospectively analyzed according to clinical and radiological criteria.

Fourteen cases showed complete alleviation of pain one week after surgery. No relapse was detected even in the subject who continued complaining of pain. All patients were treated with a day-hospital regimen and were discharged with partial weight bearing. Total weight bearing was allowed after one month, and sports were allowed after consolidation, which occurred in all but one case after the third month. One patient, who did not follow our medical advice, returned to sports activities after two weeks and experienced a fracture as a result. Atrophy of the vastus lateralis muscle developed after the procedure in another patient.

Our case series suggests that this method is reliable and safe. The level of complexity is comparable with other minimally invasive percutaneous procedures. The cost is low because there is no need to buy probes or other equipment. The negative points include weakening of the bone and the logistical problem of assembling the orthopedic surgeon, radiologist, and anesthesiologist in the tomography room.

## Introduction

An osteoid osteoma is a small, painful, benign tumor that primarily affects people younger than 30 years old. An osteoid osteoma is less than 2 cm in diameter, which distinguishes it from an osteoblastoma (Campanacci et al. 1999, Schulman & Dorfman 1970, Akhlaghpoor et al. 2007, Vanderschueren et al. 2002). The pain is not associated with physical activity, usually intensifies at night, and is typically alleviated by aspirin or other NSAIDs (Vanderschueren et al. 2002, Fenichel et al. 2006, David et al. 1997). The bones that are most affected are the femur and the tibia, accounting for 50% of the cases, although theoretically any bone could be affected (Gangi et al. 2007).

The difficulties in treating osteoid osteomas lie in the intra-surgical pinpointing of the tumor and the mechanical precariousness of the remaining bone after resection (Steinberg et al. 1990). Total resection must be achieved to avoid relapse and assure pain relief (Norman 1978). Because not all osteoid osteomas can be visualized with the image intensifier, one proposed solution was to pinpoint its location prior to resection using a computed tomography (CT) scan and mark it with a Kirschner wire, which could then be easily found at the time of surgery (David et al. 1997, Steinberg et al. 1990). Another possibility was to use a radioactive tracer with bone tropism (Pratali et al. 2009). In addition to identifying the affected area, the use of a probe during surgery allows the complete resection of the tumor to be confirmed (Pratali et al. 2009).

With respect to the reduction of morbidity caused by mechanical instability, the focus was to reduce the amount of normal bone removal without failing to remove the tumor tissue. Extensive resectioning predisposes the patient to fractures, thereby demanding long periods of rest (Rosenthal et al. 1998). Various techniques were developed to address this issue, such as the gradual elimination of the nidus by curettage or planing, by drilling with a bit, or by localized resectioning with a trephine (David et al. 1997, Ward et al. 1993, Assoun et al. 1993, Voto et al. 1990). Destruction of the nidus without resectioning by radiofrequency ablation, electro-coagulation, laser ablation, or the injection of ethanol were also described with varying incidences of relapse (Nayman et al. 2011, Mahnken et al. 2006, Busser et al. 2011, Capanna et al. 1991, Adam et al. 1995, Peyser et al. 2009, Lindner et al. 2001).

There is no consensus about the best method to treat osteoid osteomas. The technique of CT-guided percutaneous drilling is appealing because it minimizes the complications of more extensive surgeries, facilitates the pinpointing of the nidus, provokes minimum trauma in the bone and neighboring tissues, diminishes hospitalization time and causes less post-operative pain (Rosenthal et al. 1998, Peyser et al. 2009).

## Materials and methods

The medical data of 15 consecutive patients treated by CT-guided percutaneous drilling for osteoid osteoma between 2003 and 2009 were reviewed after approval of the ethics committee. Twelve patients were male, and three were female. The average age at diagnosis was 14.2 years (4 to 25 years).

**Figure 1:**
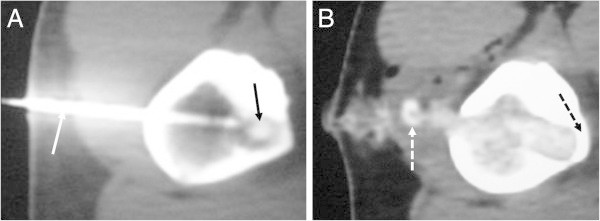
**A. CT showing the well positioned K-wire (white arrow).** Access to the nidus (black arrow) via the opposite cortex was useful in this case to avoid the neurovascular bundle. **B**. CT after resection. Note that the cortex was not totally drilled. The black dashed arrow indicates the remaining cortex. Small bone debris was left in the path and is considered harmless (dashed white arrow).

The procedure was performed in the CT room with the patient anaesthetized by spinal block and sedation. After pinpointing the nidus by tomography, the skin was marked in the area of the lesion, and the patient was slidden out from the CT arc. Based on the image and skin markings, the bone was punctured by an 11 G bone cannula. The patient was slidden back into the CT, and the positioning was confirmed. If the positioning was correct, a small fragment was removed with the cannula for a biopsy, and a Kirschner wire was introduced into the same opening and anchored in the cortical bone. Next, the bone was drilled with a 7.0 mm tubular bit, followed by a 10.0 mm rounded bit if necessary. The patient was once again slidden into the machine to confirm the complete resection of the tumor. Repositioning the guide wire and moving it a few millimeters towards the residual lesion was occasionally necessary for supplementary resectioning. Ideally, the deepest part of the cortex was preserved, but frequently the cortex has been completely severed. In some situations, to avoid neurovascular structures, access was gained through the opposite cortex, requiring the perforation of both cortices. In nine cases (60%), access to the tumor was direct, and it was operated on with only a single cortical orifice. In six cases, access was obtained from the opposite side, creating two orifices In one of them part of the thickness of the cortical wall on the affected side could be preserved (Figure 1).

The bones affected were the femur in seven cases, the tibia in six, and the iliac and the distal humerus in one each.

The diagnoses were based on clinical history aided by CT and were sometimes confirmed by magnetic resonance imaging. Cases where the diagnosis was dubious were not treated by the percutaneous drilling method.

After the procedure, the improvement in the clinical symptoms was analyzed, focusing mainly on the improvement regarding pain. The patients were evaluated at one week, two weeks, one month, three months, six months, one year, two years, and five years post-operatively. Starting in the first month, X-rays were taken of the affected area to observe the bone remodeling, which was classified into four phases. The first phase showed the empty hole where the bone was drilled, the second showed a shadow in that area, the third showed the bony callus, and the last phase showed the complete remodeling of the bone.

## Results

One week after surgery, twelve patients (80%) reported a complete absence of pain. After one month, 13 patients had no more pain. Of the two remaining patients, one reported a lessening of pain three months after the procedure, and only one case showed no improvement in the pain symptoms four years after the procedure despite X-rays and scans that demonstrated complete remodeling of the bone, no relapse, and the absence of any other lesions. All patients were treated with a day-hospital regimen and were discharged with partial weight bearing. Total weight bearing was allowed after one month, and sports were allowed after consolidation, which occurred in all but one case after the third month. Histological confirmation was possible in seven cases (41%).

**Figure 2:**
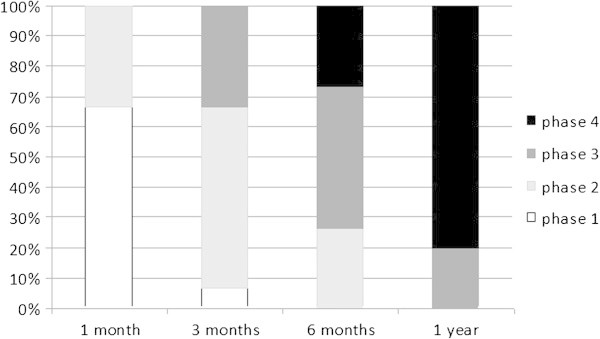
**Graph showing the percentage of patients in each phase of bone remodeling according to the follow-up time.** (Phase 1, hole; phase 2, shadow; phase 3, callus; phase 4, remodeled).

The X-rays showed rapid filling of the hole with new bone. After one month, five patients were already shown to be in phase two with incipient calluses by X-ray, and at three months, five had bony calluses blocking the hole (Figure 2).

There were two complications that had no impact on the outcome of the treatment. In one case, there was a lesion of the motor branch of the quadriceps with mild atrophy of the vastus lateralis muscle. The other case presented with an incomplete fracture in the drilled region of the tibia 13 days after the procedure. This fracture occurred during a sports activity because the patient was completely free of pain and did not follow the medical recommendation to avoid full weight bearing at that time. The use of a functional brace was enough for the patient to recover completely.

## Discussion

The rationale of osteoid osteoma treatment consists of complete resection of the nidus with minimum bone weakening. The existing methods of reaching these objectives lie between two extremes when cost and morbidity are analyzed. On one hand, there are open en block and curettage techniques. These methods have a low cost advantage and offer a confirmation of complete resection of the nidus during the procedure, combined with a low relapse rate and an anatomopathological diagnosis because the entire lesion is sent for biopsy (Campanacci et al. 1999). The disadvantages are the higher morbidity, the need for more prolonged post-operative rest periods and sometimes a greater risk of fractures (Campanacci et al. 1999, David et al. 1997, Rosenthal et al. 1998). An additional disadvantage is the difficulty in pinpointing the nidus during open resectioning, even with the use of an image intensifier (Steinberg et al. 1990).

On the other hand, there are percutaneous techniques such as radio frequency ablation, laser photo-ablation, and ethanol injection. The advantages of these methods include the ease of pinpointing the nidus with tomography and a reduced morbidity rate due to minimal resection of the bone (Akhlaghpoor et al. 2007, Vanderschueren et al. 2002, Gangi et al. 2007, Rosenthal et al. 1998, Nayman et al. 2011, Mahnken et al. 2006, Busser et al. 2011, Capanna et al. 1991, Adam et al. 1995, Peyser et al. 2009, Lindner et al. 2001). These techniques show relapse rates comparable with those of open resectioning and do not require post-operative immobilization because the patient can bear weight on the operated limb as soon as the first day after the procedure. The disadvantages include the high cost of acquiring the probes and specific materials required by these techniques and being unable to confirm the complete resection of the nidus during the procedure (Vanderschueren et al. 2002, Lindner et al. 2001). An additional disadvantage is the low rate of anatomopathological confirmation due to the scant quantity of material sent for biopsy, even though this rate can range from 36% to 90% (Fenichel et al. 2006, Gangi et al. 2007, Peyser et al. 2009, Lindner et al. 2001, Mylona et al. 2010).

The percutaneous CT-guided drilling technique presented in this study confirms this method as an appealing alternative compared with the other treatment options. It demonstrated a high rate of success (14 of 15 patients, 94%), similar to that of the other percutaneous methods (Vanderschueren et al. 2002, Gangi et al. 2007, Peyser et al. 2009). Because the entire surgery, from pinpointing the nidus to the end of the drilling, is performed with the aid of tomography, the resectioning remains controlled during the procedure with no need of a second intervention due to an incomplete resection, which can occur in the other percutaneous methods (Vanderschueren et al. 2002, Gangi et al. 2007, Peyser et al. 2009) in which the destroyed nidus cannot be visually monitored. The relapse rates are comparable with those of all the other methods. The anatomopathological confirmation rate is also comparable with that of the other percutaneous procedures (Gangi et al. 2007, Peyser et al. 2009, Lindner et al. 2001).

Drilling, which is a percutaneous technique, also does not necessitate prolonged rest time or additional implants to guarantee stability. It has a low cost because there is no need for special probes or other equipment and the hospitalization time is short. The level of complexity is comparable with that of other percutaneous procedures involving minimally invasive techniques and guided bone biopsies.

We consider a potential negative point to be the logistical problem of assembling the orthopedist, radiologist, and anesthesiologist at the same time in the tomography room. The other percutaneous treatment methods also need to use CT guiding to be more precise, and therefore this inconvenience is not unique to the described technique.

Our results confirm the high success rate and low complication rate of the described method and suggest that early weight bearing is safe. Surgeon’s selection of a procedure depends on subjective preference, because no comparative studies are currently available.
